# SARS‐CoV‐2′s claimed natural origin is undermined by issues with genome sequences of its relative strains: Coronavirus sequences RaTG13, MP789 and RmYN02 raise multiple questions to be critically addressed by the scientific community

**DOI:** 10.1002/bies.202100015

**Published:** 2021-05-27

**Authors:** Yuri Deigin, Rossana Segreto

**Affiliations:** ^1^ Youthereum Genetics Inc. Toronto Ontario Canada; ^2^ Department of Microbiology University of Innsbruck Innsbruck Austria

**Keywords:** furin cleavage site, origin, Pangolin CoV MP789, peer review, SARS‐CoV‐2, RmYN02, RaTG13

## Abstract

RaTG13, MP789, and RmYN02 are the strains closest to SARS‐CoV‐2, and their existence came to light only after the start of the pandemic. Their genomes have been used to support a natural origin of SARS‐CoV‐2 but after a close examination all of them exhibit several issues. We specifically address the presence in RmYN02 and closely related RacCSxxx strains of a claimed natural PAA/PVA amino acid insertion at the S1/S2 junction of their spike protein at the same position where the PRRA insertion in SARS‐CoV‐2 has created a polybasic furin cleavage site. We show that RmYN02/RacCSxxx instead of the claimed insertion carry a 6‐nucleotide deletion in the region and that the 12‐nucleotide insertion in SARS‐CoV‐2 remains unique among Sarbecoviruses. Also, our analysis of RaTG13 and RmYN02's metagenomic datasets found unexpected reads which could indicate possible contamination. Because of their importance to inferring SARS‐CoV‐2′s origin, we call for a careful reevaluation of RaTG13, MP789 and RmYN02 sequencing records and assembly methods.

## Graphical abstract

One of the main arguments supporting a natural origin for SARS‐CoV‐2 is the identification of genetically close coronavirus sequences (RaTG13, MP789, RmYN02) which share some of its peculiar features. However, a close examination reveals that all of them exhibit several issues which should be re‐evaluated in an open and critical review process.

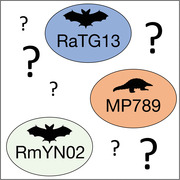

## INTRODUCTION

SARS‐CoV‐2 has drastically changed the world, causing catastrophic loss of life and immense economic disruption. Establishing its origins is therefore of utmost importance, but more than a year since the outbreak in Wuhan, the scientific community has yet to provide a definitive answer. The search for SARS‐CoV‐2′s origins in nature relies on finding closely related coronavirus (CoV) sequences in primary or secondary hosts, as a possible source of zoonotic spill‐over to humans. RaTG13,^[^ ^1^ ^]^ MP789,^[^ ^2^ ^]^ and RmYN02^[^ ^3^ ^]^ are among the CoVs most closely related to SARS‐CoV‐2 identified so far, and their existence came to light only after the beginning of the pandemic. Countless scientific publications refer to these key sequences in their attempts of inferring SARS‐CoV‐2′s origin. Upon close examination, all three of these sequences and/or the papers where they have been first described are flawed by several issues that should be carefully addressed by the scientific community.

## THE ADDENDUM TO THE PAPER DESCRIBING FIRST RATG13 OPENS MORE QUESTIONS THAN THE ONES ANSWERED

Shortly after the beginning of the pandemic, Zhou et al.^[^ ^1^ ^]^ have published a key paper first describing RaTG13, which is SARS‐CoV‐2′s closest relative found so far (96.2% identity). Very little information on sampling site and sequencing methods was released by the authors at the time. Zhou et al.^[^ ^1^ ^]^ stated: ”We then found that a short region of RNA‐dependent RNA polymerase (RdRp) from a bat coronavirus (BatCoV RaTG13)—which was previously detected in *Rhinolophus affinis* from Yunnan province—showed high sequence identity to 2019‐nCoV. We carried out full‐length sequencing on this RNA sample.” Intriguingly, in the preprint^[^ ^4^ ^]^ for the above article, the quoted sentence originally said “which we previously detected” rather than “which was previously detected”. It is unclear why the authors chose to further distance themselves from the collection of RaTG13 in the final version of their paper.

After repeated requests for clarifications from several scientists and journalists and more than 9 months later, the Zhou et al.^[^ ^1^ ^]^ paper has been amended with an Addendum,^[^ ^1^ ^]^ which provides some missing information on RaTG13, most of it previously discovered and made public by an independent research group named “DRASTIC”^[^ ^5^ ^]^ and published by Rahalkar and Bahulikar^[^ ^6^ ^]^ and Segreto and Deigin.^[^ ^7^ ^]^

While the Addendum clarifies some crucial points, such as exact sampling location of RaTG13, and mentions the original paper describing it,^[^ ^8^ ^]^ the information released is still incomplete and partially in conflict with data previously provided. In this regard, the Addendum clarifies that RaTG13 has been fully sequenced in 2018^[^ ^5^ ^]^ and not after the beginning of the pandemic, as seemingly implied by Zhou et al.^[^ ^1^ ^]^ as a result of them having matched SARS‐CoV‐2 to that short RdRp region. It is important to notice that if the full genome of RaTG13 was present in their database since 2018, it would have immediately come up as the best match to SARS‐CoV‐2 when queried in 2020, with no need to mention the match to its short RdRp region.

Moreover, the Addendum confirms our suggestion^[^ ^7^ ^]^ that RaTG13's partial RdRp mentioned by Zhou et al.^[^ ^1^ ^]^ could have been previously named RaBtCoV/4991,^[^ ^8^ ^]^ which is a sample collected in 2013 in a mine where six workers—three of whom died—contracted pneumonia with very similar symptoms as SARS‐CoV‐2, and later four of whom were confirmed by WIV to carry antibodies against SARS.^[^ ^6^, ^7^ ^]^

It should be mentioned that the peer‐review process of the Zhou paper^[^ ^1^ ^]^ failed to ensure that the authors numerically define their stated “high sequence identity” of RaTG13's partial RdRp to SARS‐CoV‐2, as instead was done by Chen et al.^[^ ^9^ ^]^ in a paper submitted in the same period, which reports 98.7% identity of RaBtCoV/4991 to SARS‐CoV‐2 MN988668 and MN988669.

In addition, new information revealed by the Addendum is that eight other beta‐SARSr‐CoVs distantly related to SARS‐CoV were also isolated from the same Mojiang mine, and sequenced together with RaTG13, but neither their genomes, nor information about their sample names and eventual accession numbers is provided. It is not known how these sequences relate to RaTG13. The Addendum also fails to release details about the number and kind of samples collected from the mine workers, their storage conditions, methods used for each test described and specification of the results obtained.

In addition, the Addendum fails to address and/or contradicts the statements in an MSc^[^ ^10^ ^]^ and a PhD^[^ ^11^, ^12^ ^]^ theses which have previously described in detail the miners’ pneumonia symptoms and stated that SARS Immunoglobulin G (IgG) antibodies were detected by the Wuhan Institute of Virology (WIV) in all four of the miners’ samples tested.

Various preprints^[^ ^13^, ^14^, ^15^, ^16^ ^]^ have questioned the validity of the metagenomic dataset upon which RaTG13 is based. For an independent analysis of the raw data used for RaTG13's assembly, we ran NCBI BLAST (blastn suite) using RaTG13 (MN996532.2) as query sequence against RaTG13's raw reads (SRX7724752) and amplicons (SRX8357956). The first 14 nucleotides (nt) of the 5′ end of RaTG13 had no sequence matches, which is unexpected not only because the Genbank entry for RaTG13 has been edited on 13 October 2020^[^ ^17^ ^]^ and the 5′ end was added without support from raw data, but also because the sample was stated to have been fully depleted during its sequencing carried out in 2018.^[^ ^18^ ^]^ In the same update, a small number of nucleotides were also edited, possibly fixing assembly errors of the first genome release. As all these modifications were introduced without explanations and without uploading further sequencing data, we call for information on the assembly process of the first RaTG13 genome to be released together with the reads supporting the bases that contradict sequencing data.

To verify the criticisms expressed about RaTG13's low number of bacterial reads being unexpected for a fecal swab, we performed a taxonomic analysis of the raw reads using the NCBI SRA Taxonomy Analysis Tool. Only 0.65% of the raw reads were composed of bacteria and a significant quantity of sequences unexpectedly belonged to species with habitats well outside of Yunnan Province, China (4.6% *Rousettus aegyptiacus*; 4.6% *Marmota marmota marmota*; 3.6% *Marmota flaviventris*). The anomalously low bacterial quantity is striking when compared with the raw reads from *Rhinolophus affinis*’s fecal swab (SRR11085736) uploaded to Genbank by the WIV on the same day as RaTG13's dataset (13 February 2020) and which contains 91% bacteria.

Zhang^[^ ^13^ ^]^ and Singla^[^ ^15^ ^]^ further identified in RaTG13's raw reads the presence of uncommonly abundant telomere‐like sequences. Telomeres are DNA‐protein structures composed of tandem repeats which are located at the end of chromosomes and usually represent only a minor fraction of total cellular RNA extracted from a biological sample. We calculated with TelomereCat^[^ ^19^ ^]^ that the RaTG13 raw reads (Genbank accession SRX7724752) are composed of 14% fully telomeric sequences. The origin of these repeats is unexplained and a more thorough investigation of telomere‐like sequences in the dataset is warranted.

We then ran BLASTn for randomly selected raw reads from RaTG13's dataset against the NCBI Nucleotide Collection Database using a minimum similarity of 95% until we recorded 1698 hits. Surprisingly, 10% of the sequences identified matched the *Homo sapiens* genome, indicating significant contamination of RaTG13's dataset, which might have happened during sequencing or purification from human cell cultures.

Considering that RaTG13 has been presented as evidence that SARS‐CoV‐2 may have naturally originated in bats^[^ ^1^ ^]^ and that it shares many novel features with SARS‐CoV‐2′s genome—among them the presence of multiple inserts in the spike protein^[^ ^1^ ^]^—it should not be used to draw conclusions about SARS‐CoV‐2′s natural origin until its reliability is proven.

## THE SAME PANGOLIN CORONAVIRUS SEQUENCE MP789 HAS BEEN CITED BY SEVERAL PUBLICATIONS UNDER DIFFERENT NAMES

The identification of an RBD very similar to the one present in SARS‐CoV‐2 in CoV isolated from a batch of pangolins smuggled from the Guangdong province (GD, China) in March 2019^[^ ^2^ ^]^ have raised speculations that pangolins could have been a possible host for SARS‐CoV‐2 before its jump to humans, although its overall genome similarity is lower to SARS‐CoV‐2 than that of RaTG13.^[^ ^20^ ^]^ Upon close examination of the assembled genomes and raw data, Chan and Zhan^[^ ^21^ ^]^ have discovered that this particular RBD was found only in two pangolin samples out of 13 collected (#7 and #8) and that the same resulting assembled genome has been differently named by Liu et al.^[^ ^2^ ^]^ and Xiao et al.^[^ ^20^ ^]^ (respectively MP789 and GD_1). Considering the rarity of this special RBD in the pangolin samples analyzed, Chan and Zhan^[^ ^21^ ^]^ conclude that pangolins could have been infected by other animals during trafficking and other authors even suggest possible contamination of the pangolin dataset by human sequences^[^ ^22^ ^]^ or cell cultures.^[^ ^23^ ^]^ Based on these findings, the “U.S. Right to Know” association has requested detailed clarifications^[^ ^24^ ^]^ on the pangolin dataset from the authors Liu et al.^[^ ^2^ ^]^ and Xiao et al.,^[^ ^20^ ^]^ and the editors of *PLoS Pathogens* and *Nature*, which have published several papers based on the same dataset.^[^ ^25^, ^26^ ^]^

Many questions still await an answer but as a result of the inquiry a note has been added to Xiao et al.,^[^ ^20^ ^]^ alerting readers about the sample's ongoing issues:

“Editor's Note: Readers are alerted that concerns have been raised about the identity of the pangolin samples reported in this paper and their relationship to previously published pangolin samples. Appropriate editorial action will be taken once this matter is resolved.”

However, several papers have already relied on MP789 for their analysis, namely the widely cited “The Proximal Origin of SARS‐CoV‐2″ paper published in Nature Medicine by Andersen et al.^[^ ^27^ ^]^ that concludes that SARS‐CoV‐2 most likely originated in nature. Recent analyses have questioned the possibility of pangolins as possible intermediate hosts for SARS‐CoV‐2,^[^ ^28^, ^29^ ^]^ therefore Andersen et al.^[^ ^27^ ^]^ and other authors relying on MP789 should carefully re‐evaluate their conclusions. SARS‐CoV‐2′s RBD, which appears to be highly adapted to human ACE2^[^ ^30^ ^]^—even more than the one developed by severe acute respiratory syndrome (SARS‐CoV) in 2002/2003,^[^ ^31^ ^]^ remains a very peculiar feature.

## THE CLAIMED PAA/PVA INSERTION IN RmYN02/RacCSxxx STRAINS IS HIGHLY DOUBTFUL

Zhou et al.^[^ ^3^ ^]^ reported the discovery of a novel CoV strain RmYN02, which the authors claim to contain a natural PAA amino acid insertion at the S1/S2 junction of the spike protein at the same position as the PRRA insertion which has created a polybasic furin cleavage site (FCS) in SARS‐CoV‐2. Likewise, the same group of authors has also recently labeled as an insertion a very similar PVA fragment in a newly reported cluster of Thai CoVs (RacCS203, RacCS264, RacCS271, collectively referred to as RacCSxxx hereinafter).^[^ ^32^ ^]^

Zhou et al.^[^ ^3^ ^]^ have come to their conclusion based on a multiple sequence alignment of RmYN02 with several beta coronavirus strains, namely SARS‐CoV‐2, SARS‐CoV GZ02, RaTG13, ZC45, ZXC21, Pangolin/GD/2019 (MP789), and Pangolin/GX/P5L/2017. Their findings are reported in a single amino acid alignment diagram where the supposed PAA amino acid insertion is placed between the 680 (serine) and 685 (arginine) amino acids of SARS‐CoV‐2′s spike protein. The authors do not provide details about the algorithm applied to obtain the alignment and if alternative alignments were generated during their analysis. Considering that no single algorithm can always achieve the best alignment for a given dataset,^[^ ^33^ ^]^ conclusions should be drawn based on several alignment methods, as well as validation of the results by a trained human eye.

**FIGURE 1:**
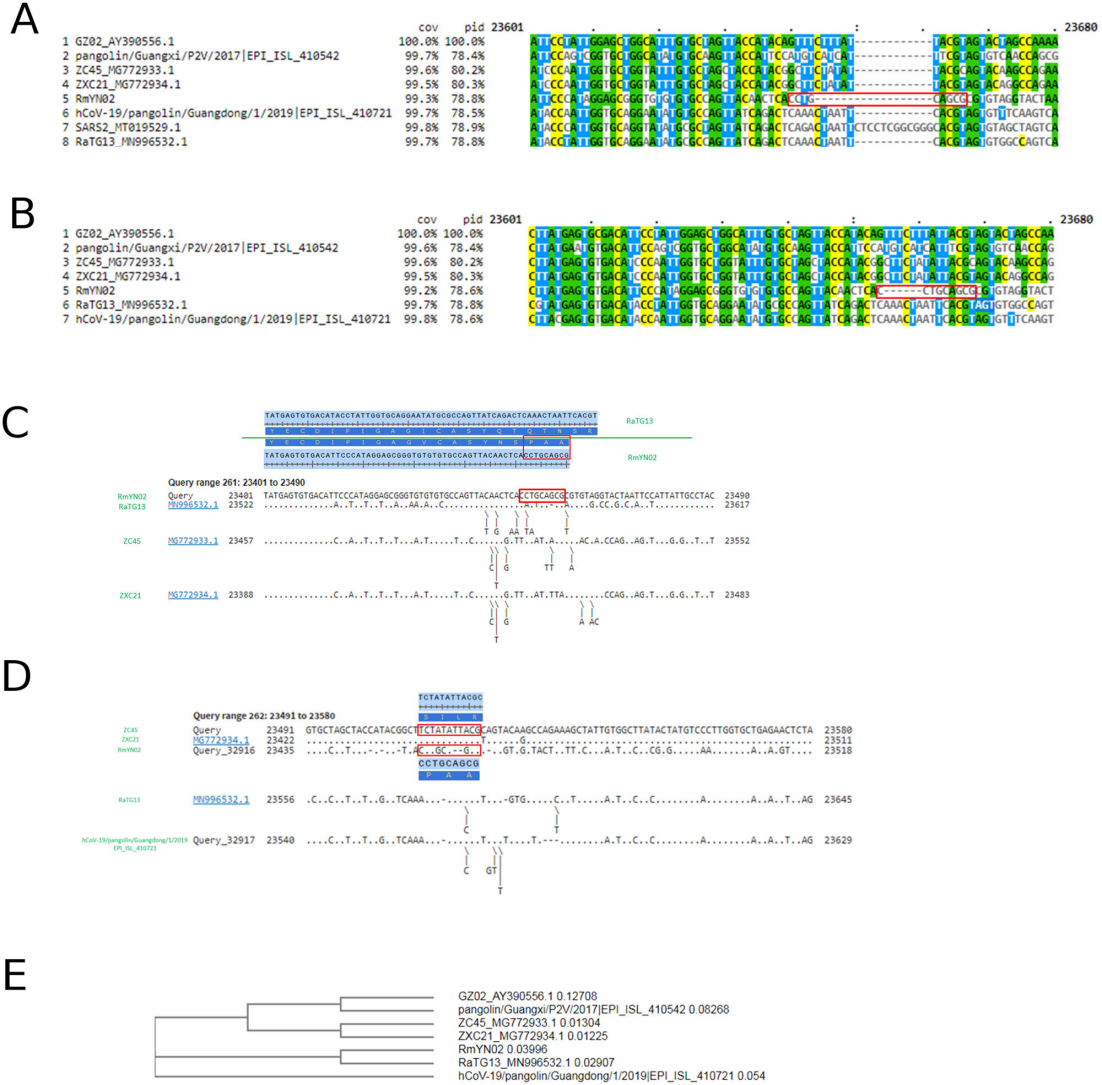
(A) Clustal W multiple sequence alignment of RmYN02 with the strains used in Zhou et al. for comparison. RmYN02's nucleotides coding the PAA amino acids (CCT GCA GCG) are surrounded by a red box. No insertion in RmYN02 is visible; on the contrary, a deletion splitting the nucleotides coding for PAA is observed. (B) Clustal W multiple sequence alignment of RmYN02 with the strains used in Zhou et al. for comparison, with the exception of SARS‐CoV‐2. The deletion characterizing RmYN02 at the S1/S2 junction appears to cause a split of the first nucleotide from the rest of the sequence coding for the PAA amino acids (CCT GCA GCG, surrounded by a red box). (C) Pairwise comparisons of RmYN02 (anchor) to RaTG13, ZC45, and ZXC21. No PAA insertion is observed in RmYN02 in these comparisons. (D) Pairwise comparisons of ZC45 (anchor) to ZXC21, RmYN02, RaTG13, and Pangolin/GD/2019. RmYN02's nucleotides coding the PAA amino acids (surrounded by a red box) are aligned as mutations relative to ZC45 rather than insertions. (E) Phylogenetic tree of SARS‐GZ02, Pangolin/GX/2017, ZC45, ZXC21, RmYN02, RaTG13, and Pangolin/GD/2019 produced by CLUSTAL W based on the alignment of their genomes as in (B)

Moreover, no nucleotide alignment of the same region is provided by Zhou et al.^[^ ^3^ ^]^ that could allow the reader to identify the underlying nucleotides (CCT GCA GCG) coding for the claimed PAA insertion in RmYN02 in relation to the other strains analyzed. We have thus performed a CLUSTAL W^[^ ^34^ ^]^ multiple nucleotide sequence alignment of the strains reported in Zhou et al.^[^ ^3^ ^]^ but were unable to observe the claimed insertion (Figure 1A). RmYN02 instead appears to contain a 6‐nucleotide deletion at the S1/S2 junction when compared to the other strains, and the only insertion observed when aligning the same genomes as used by Zhou et al.^[^ ^3^ ^]^ is the well‐known 12‐nucleotide insertion CT CCT CGG CGG G (PRRA) in SARS‐CoV‐2. The 6‐nucleotide deletion in RmYN02 at the S1/S2 junction is even more apparent when SARS‐CoV‐2 is excluded from the multiple sequence alignment (Figure 1B).

We believe that including SARS‐CoV‐2 in the input to a multiple alignment algorithm together with RmYN02 and other strains, as Zhou et al.^[^ ^3^ ^]^ have done, is methodologically incorrect, because the implied underlying hypothesis which their analysis is meant to test is whether SARS‐CoV‐2′s PRRA insertion is of natural origin. Thus, including SARS‐CoV‐2 in the alignment not only biases the alignment algorithm, but also pre‐supposes the conclusion that the PRRA insert is, indeed, natural. To prove that inserts like PRRA occur naturally, strains that exhibit similar inserts must be compared to their relative strains, excluding SARS‐CoV‐2 from the analysis.

Our analyses show that RmYN02 does not contain an insertion at the S1/S2 junction when compared to its closest relatives and the claimed PAA insertion is more likely to be the result of mutations. Pairwise comparisons between RmYN02 and its closest relatives (RaTG13, ZC45, ZXC21) confirm this hypothesis when either RmYN02 (Figure 1C) or ZC45 (Figure 1D) are used as an anchor, and instead produce a 2‐nt deletion in the coding region for PAA (Figure 1D). If RmYN02 truly had an insertion comparable to the PRRA insertion in SARS‐CoV‐2, we would have expected such an insertion to be clearly observable in pairwise comparisons to RmYN02's closest relatives, such as RaTG13, ZC45, ZXC21, and Pangolin/GD/2019 (Figure 1E).

**FIGURE 2:**
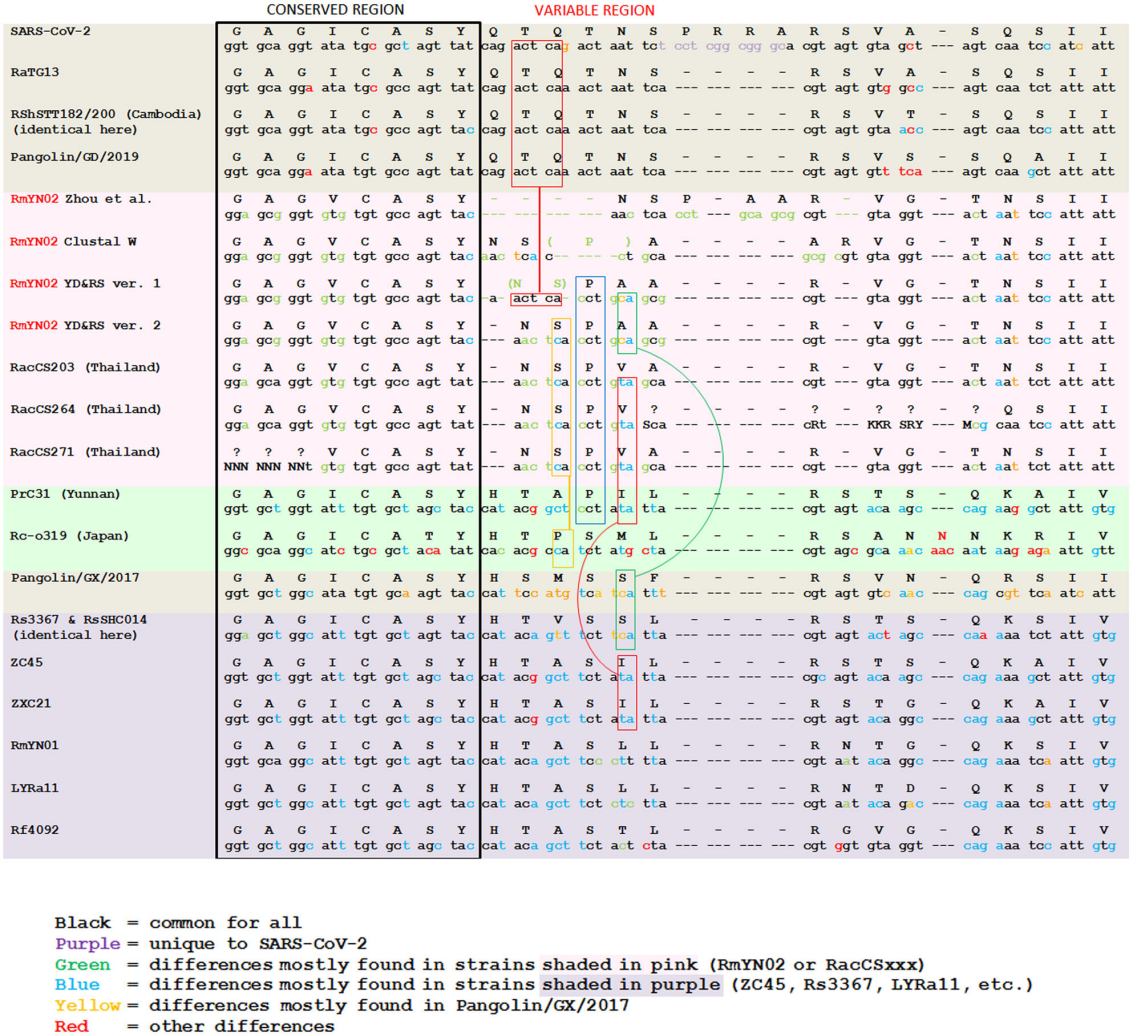
Nucleotide and amino acid alignments of RmYN02 with SARS‐CoV‐2, RaTG13, RShSTT182/200 (Cambodia), RacCS203/264/271 (Thailand), Pangolin/GD/2019, RmYN01, RP3, Rf4092, LYRa11, Rs3367, RsSHC014, ZC45, and ZXC21 at the S1/S2 junction of the spike protein. For RmYN02, three alternative versions are provided, besides the ones proposed by Clustal W and Zhou et al

A close examination of the S1/S2 region reveals that in RmYN02 it is six nucleotides (two amino acids) shorter than those of its related strains RaTG13, Pangolin/GD/2019, ZC45, and ZXC21. Therefore, to support the claimed PAA insertion not only a 9‐nucleotide insertion, but also a 15‐nucleotide deletion must have occurred. While this is theoretically possible, we propose two alternatives of more parsimonious alignments which do not have any insertions (versions 1 and 2 in Figure 2). The alignment proposed by CLUSTAL W (Figure 1B) also did not produce any insertions (ver. “Clustal W” in Figure 2).

Rather than a complete 12‐nucleotide deletion of the region in RmYN02 that corresponds to QTQT in RaTG13 as proposed by Zhou et al.,^[^ ^3^ ^]^ we believe a more parsimonious scenario is just a 3‐nucleotide deletion: either just a deletion of the first Q codon (version 2 in Figure 2) or a discontinuous 3‐nt deletion split among the nucleotides coding for QTQ that preserves in RmYN02 and RacCSxxx the continuous span of ACTCA nucleotides from their relative strains but turns QTQ into NS (version 1 in Figure 2, preserved nucleotides are marked by the topmost red box). Such deletions could result from RdRp stutter and could be tolerated as long as they do not shift the coding frame.

Another possibility, proposed by CLUSTAL W, is a 6‐nucleotide deletion in the middle of the nucleotides coding for QTN, turning it into a P. However, we view this proposed alignment as unlikely because the P (coded by CCT) in RmYN02 and RacCSxxx anchors well to the P (also coded by CCT) in the PrC31 (EPI_ISL_1098866) strain (marked by a blue box in Figure 2).

The amino acid I (coded by ATA) following P in PrC31 also aligns well with the amino acid V (coded by GTA) following P in the RacCSxxx strains. The same amino acid I (coded by ATA) is also observed in the ZC45 and ZXC21 strains in the identical position (marked by the bottom red boxes in Figure 2).

Similarly, the amino acid A (coded by GCA) following P in RmYN02 aligns well with the amino acid S (coded by TCA) in the Pangolin/GX/2017, Rs3367, and RsSHC014 strains (marked by the green box in Figure 2).

Finally, preceding the P amino acid of the PAA/PVA fragments in RmYN02/RacCSxxx is the amino acid S (coded by TCA) which aligns well with the amino acid P (coded by CCA) in the Rc‐o319 strain (marked by a yellow box in Figure 2).

A conclusive proof of any novel insertion is the existence of closely related strains without it. In the case of SARS‐CoV‐2, the PRRA insertion is obvious because closely related strains RaTG13 or Pangolin/GD/2019 do not have the PRRA fragment while still having the nearly identical nucleotides around the same locus where SARS‐CoV‐2 has the insertion. In the case of RmYN02/RacCSxxx, the purported PAA/PVA insertion is always coupled with a purported 4 amino acid deletion just preceding the NSPAA/NSPVA fragment. This deletion corresponds to a QTQT fragment in SARS‐CoV‐2, RaTG13 and Pangolin/GD/2019. If PAA/PVA truly was an insertion, one would expect to see closely related strains that do not yet have that insertion but already have the purported 4 amino acid deletion. In the absence of such strains, the more parsimonious explanation for the PAA/PVA fragments is not a 3‐aa insertion combined with a 4‐aa deletion, but point mutations and a 1‐aa deletion instead.

Taken together, the above observations conclusively show that the PAA/PVA fragments in RmYN02/RacCSxxx do not represent novel insertions but instead align well to existing PIL/SIL fragments in closely related strains, and no alignment of RmYN02 or RacCSxxx produces anything that might support the hypothesis proposed by Zhou et al.^[^ ^3^ ^]^ of a combined 15‐nt deletion and 9‐nt insertion in RmYN02/RacCSxxx.

As an aside, we would like to hypothesize that the observed 6‐nucleotide deletion at the S1/S2 junction in RmYN02 and Thai CoV RacCSxxx strains might not be a deletion *per se*, but instead an ancestral feature, and it could be the other strains, which are 6‐nt longer here, who have had their ancestor(s) develop a 6‐nt insertion at this locus.

While further virus collecting expeditions might produce unanticipated discoveries, to date SARS‐CoV‐2 remains unique among its Sarbecovirus relatives not only due to a polybasic furin site at the S1/S2 junction, but also due to the length of the locus surrounding the 12‐nucleotide insertion that has created the furin site: SARS‐CoV‐2 is at least 12 nucleotides longer at that junction than any of its Sarbecovirus relatives. Its PRRA insertion is beyond any doubts, and was not accompanied by any deletions, which stands in sharp contrast to what is observed in RmYN02. We demonstrated that RmYN02 cannot be used to support a natural origin of the furin cleavage site in SARS‐CoV‐2, and as a consequence of SARS‐CoV‐2 itself, as concluded by Zhou et al.^[^ ^3^ ^]^

To verify the observation of Signus^[^ ^16^ ^]^ of the unusually high content of a single 3′‐ETS (External Transcribed Spacer, a piece of non‐functional RNA) sequence from *Homo sapiens* in the meta‐transcriptomic sequencing dataset used for RmYN02's assembly (SRR12432009), we ran BLASTn for randomly selected raw reads from SRR12432009 against the NCBI Nucleotide Collection Database using a minimum similarity of 95% until we recorded 4428 hits. Surprisingly, we found that 75% of the reads matched the Genbank sequence “*Homo sapiens* external transcribed spacer 18S ribosomal RNA gene”, while 2.5% matched *Chiroptera* or bat CoV sequences. The dominant presence of a single human RNA gene in the dataset used for RmYN02's assembly suggests that also RmYN02's metagenomic dataset is clearly contaminated, as found for RaTG13, and it should not be relied upon for research purposes until verified.

In closing, we would like to point out another improper alignment in the Zhou et al.^[^ ^32^ ^]^ preprint: in Fig. 4, the authors mistakenly shift the RSANNN fragment of Rc‐o319 by one amino acid to the left, aligning it with the ARSVAS fragment of SARS‐CoV‐2. However, as a quick look by a trained eye at the underlying nucleotides will show, the RSANNN fragment of Rc‐o319 best aligns with the RSVN‐Q of Pangolin/GX/2017 in the same Fig. 4. Further proof of this alignment is provided by PrC31, Rs3367, and RsSCH014 in our analysis (Figure 2).

One final minor point that we would like to make is that RmYN02's assembled sequence is presently only available in the GISAID database, which is password protected and requires registration. We would propose that RmYN02 should also be made available at GenBank.

## CONCLUSION

RaTG13, MP789 and RmYN02 are among SARS‐CoV‐2′s closest relatives and therefore of utmost importance as key tools for inferring SARS‐CoV‐2′s phylogenetic relationships and to identifying SARS‐CoV‐2′s specific genetic features, with the final aim of uncovering its origin. These sequences have been widely used to support a natural origin of SARS‐CoV‐2 but after a close examination, all of them exhibit issues which should be specifically addressed and clarified. It should be also noted that amplicon and raw data connected to these sequences have been made available only after request from scientists willing to verify the assembled published genomes. Lack of accuracy and missing or conflicting information in the papers describing these key sequences should have been resolved during a thorough peer review process. Considering the criticisms expressed by several researchers about these sequences and related papers, alternative analyses based only on sequences released before the beginning of the pandemic should be taken into account when drawing conclusions about SARS‐CoV‐2′s origin. In conclusion, we propose that the review process of all papers describing SARS‐CoV‐2′s closest relatives which could contribute to identify SARS‐CoV‐2′s origin should made public, allowing an open and critical evaluation by the entire scientific community.

## CONFLICT OF INTEREST

The author declares no conflict of interest.

## DATA AVAILABILITY STATEMANT

*Source code for all analyses can be* found at https://github.com/bioscienceresearch/Genome_Sequence_Reliability
